# Practical management of disease-related manifestations and drug toxicities in patients with multiple myeloma

**DOI:** 10.3389/fonc.2024.1282300

**Published:** 2024-03-22

**Authors:** Catarina Geraldes, Adriana Roque, Ana Bela Sarmento-Ribeiro, Manuel Neves, Alina Ionita, Rita Gerivaz, Ana Tomé, Sofia Afonso, Maria Pedro Silveira, Patrícia Sousa, Rui Bergantim, Cristina João

**Affiliations:** ^1^ Serviço de Hematologia Clínica, Centro Hospitalar e Universitário de Coimbra, Coimbra, Portugal; ^2^ Faculty of Medicine, University of Coimbra, Coimbra, Portugal; ^3^ Coimbra Institute for Clinical and Biomedical Research, Faculty of Medicine, University of Coimbra, Coimbra, Portugal; ^4^ Center for Innovative Biomedicine and Biotechnology (CIBB), University of Coimbra, Coimbra, Portugal; ^5^ Clinical Academic Center of Coimbra (CACC), Coimbra, Portugal; ^6^ Institute of Physiology, Faculty of Medicine, University of Coimbra, Coimbra, Portugal; ^7^ Hemato-Oncology Unit, Champalimaud Foundation, Lisboa, Portugal; ^8^ Hematology Department, Portuguese Institute of Oncology Francisco Gentil, Lisboa, Portugal; ^9^ Serviço de Hemato-oncologia, Hospital Garcia de Orta, Lisboa, Portugal; ^10^ Serviço de Hematologia Clínica, Centro Hospitalar Universitário Cova da Beira, Covilhã, Portugal; ^11^ Faculdade de Ciências da Saúde, Universidade da Beira Interior, Covilhã, Portugal; ^12^ Serviço de Imuno-Hemoterapia, Hospital Prof. Doutor Fernando Fonseca, EPE, Amadora, Portugal; ^13^ Haematology Department, Portuguese Oncology Institute of Coimbra, Coimbra, Portugal; ^14^ Serviço de Hematologia Clínica, Centro Hospitalar Universitário de São João (CHUSJ), Porto, Portugal; ^15^ Instituto de Investigação e Inovaçáo em Saúde, Universidade do Porto, Porto, Portugal; ^16^ Institute of Pathology and Molecular Immunology, Abel Salazar Institute of Biomedical Sciences, University of Porto, Porto, Portugal; ^17^ NOVA Medical School, Universidade Nova de Lisboa, Lisboa, Portugal

**Keywords:** delivery of health care, cancer management, anticancer drug toxicities, multiple myeloma, real life practical considerations, myeloma-related manifestations

## Abstract

Multiple myeloma (MM) is a very heterogeneous disease with multiple symptoms and clinical manifestations. MM affects mainly elderly patients and is difficult to manage in the presence of comorbidities, polypharmacy, frailty and adverse events of disease-targeted drugs. The rapid changes in MM treatment resulting from constant innovations in this area, together with the introduction of numerous new drugs with distinct mechanisms of action and toxicity profiles, have led to an increased complexity in the therapeutic decision-making and patient management processes. The prolonged exposure to novel agents, sometimes in combination with conventional therapies, makes this management even more challenging. A careful balance between treatment efficacy and its tolerability should be considered for every patient. During treatment, a close monitoring of comorbidities, disease-related manifestations and treatment side effects is recommended, as well as a proactive approach, with reinforcement of information and patient awareness for the early recognition of adverse events, allowing prompt therapeutic adjustments. In this review, we discuss various issues that must be considered in the treatment of MM patients, while giving practical guidance for monitoring, prevention and management of myeloma-related manifestations and treatment-related toxicities.

## Introduction

Multiple myeloma (MM) represents the second most frequent hematologic malignancy, with more than 176,000 new cases diagnosed worldwide and more than 50,000 in Europe in 2020 (1). It accounts for 1% of all cancers and approximately 10% of all hematologic malignancies.

In the last 2 decades, remarkable advances in the treatment of patients with MM have resulted in a significant improvement in prognosis, with increased overall survival (2), and have converted MM into a chronic disease in a considerable number of cases (3). New therapeutic agents with more selective targeted mechanisms of action and inducing less acute toxicity, when compared to classical cytotoxic agents, introduced the concept of continuous therapy and maintenance therapy. The current MM treatment paradigm includes combinations of antineoplastic drugs until disease progression, with patients continuously maintaining the same drug regimen for several years (4).

MM is a neoplasm that mostly affects elderly patients, with a median age at the time of diagnosis of 65 years (5). Consequently, many of these patients are frail,have age-related comorbidities at diagnosis or during the course of the disease, and are likely to experience polypharmacy (6). The most common comorbidities at diagnosis are cardiovascular disease (63-69%) (7), diabetes mellitus or other disorders of glucose metabolism (11-22% of patients with MM) (8), hypertensive, diabetic or atherosclerotic nephropathy(up to 15% of cases at presentation) (9, 10), neurological and cognitive changes, osteoarticular disease, poor bone marrow reserve and immunosenescence (5).

The patient population treated in real-life clinical practice differs from the patient population included in clinical trials, who are usually younger, with better performance status (PS) and without cardiovascular, renal or hepatic dysfunction (9). The available data on the efficacy and safety of new therapeutic agents are relatively scarce in subgroups of patients with specific comorbidities, and often fail to adequately capture long-term toxicities. The use of non-uniform criteria and definitions for frailty status and organ damage/dysfunction has implications for the quality and applicability of the scientific evidence, making it difficult to create clear recommendations that include the widest possible range of patient subgroups.

This increasing complexity poses challenges for the medical community treating these patients. The appropriate management of toxicities and comorbidities is essential to avoid treatment discontinuation or dose reduction, and the consequent compromise of treatment efficacy, as well as to optimize patients’ quality of life.

Below, we describe the adverse events, complications and comorbidities that are most frequently observed in patients with MM, during treatment with the new therapeutic combinations, as well as some recommendations for their approach.

## Infections

Patients with MM are more susceptible to infections due to disease-related factors (suppression of cellular and humoral immunity, including hypogammaglobulinemia lymphocyte dysfunction, low CD4+ cell count and impaired function of natural killer cells), patient-related factors (old age, poor performance status, and comorbidities, including diabetes mellitus and renal dysfunction) and treatment-related factors (neutropenia) (11, 12) especially within the first year of diagnosis (11). Of note, patients with a high tumor burden (International Staging System II-III), marked elevation of serum lactate dehydrogenase, poor PS, low hemoglobin and renal dysfunction are at higher risk of early and severe infections (12). The incidence of severe infection in MM patients appears to be higher during the first months after diagnosis, so the use of risk scores may help to identify patients at higher risk of infection and who may therefore benefit from individualized prophylactic treatment. A recently published score identifies serum albumin ≤30 g/L, Eastern Cooperative Oncology Group performance status (ECOG PS)>1, male gender and non-IgA type MM, as variables associated with increased risk of severe infection in the first 4 months (13, 14). In this context, infections should be approached based on the individual risk of infection (**Table 1**).

**Table 1:** Risk-adapted prophylaxis in patients with multiple myeloma.

Type of prophylaxis	Low risk	Intermediate risk	High risk
Anti-bacterial *	not recommended	Levofloxacin 500 mg qd	Levofloxacin 500 mg qd
Anti-fungal	not recommended	Fluconazole or micafungin in case of severe mucositis and/or prolonged neutropenia (ANC ≤100 cells/µL for≥7 days) Cotrimoxazole in patients on high-dose dexamethasone (doses ≥40mg x 4 days per week) or with RRMM; dapsone or atovaquone as an alternative to cotrimoxazole (prophylaxis of Pneumocystis jirovecii infection)	Fluconazole or micafungin in case of severe mucositis and/or prolonged neutropenia (ANC ≤100 cells/µL for≥7 days) Voriconazole or posaconazole if ANC ≤100 cells/µL for 7 days) Cotrimoxazole in patients on high-dose dexamethasone (doses ≥40mg x 4 days per week) or with RRMM; dapsone or atovaquone as an alternative to cotrimoxazole (prophylaxis of Pneumocystis jirovecii infection)
Anti-viral	not recommended (unless previous infection with HSV - use oral acyclovir)	Oral acyclovir - 400 mg BID (HSV); 800mg BID (HZV) or oral valacyclovir - 500 mg BID Tenofovir or entecavir (instead of lamivudine) in patients at intermediate or high risk of hepatitis B reactivation.	Oral acyclovir - 400 mg BID (HSV); 800mg 2id (HZV) or valacyclovir - 500 mg BID Tenofovir or entecavir (instead of lamivudine) in patients at high risk of hepatitis B reactivation.

HSV, Herpes simplex virus; HZV, Herpes Zoster virus; RRMM, relapsed/refractory multiple myeloma; ANC, absolute neutrophil count; qd, once a day; td, twice a day.

*Adapted to the bacterial profile of each healthcare institution/country.

The most frequent viral infections are respiratory, most often by herpes viruses (CMV, varicella zoster or herpes simplex) in patients treated with bortezomib or with monoclonal antibodies and without antiviral prophylaxis. Invasive fungal infections have an incidence of about 2.5% and the median time from diagnosis to presentation of invasive fungal infection is 35 months (15, 16). Bacterial infections are most often caused by coagulase-negative staphylococci (48%), *Enterococcus faecalis* (11%), and *Escherichia coli* (16%), among others, although bacterial strains are determined by the local epidemiology. In patients with IgG < 400 mg/dL and recurrent infections with encapsulated bacteria, or in those with proven specific antibody failure (PSAF), polyclonal immunoglobulin replacement (200-400 mg/kg IV every 3-4 weeks) may be considered (15). The International Myeloma Working Group (IMWG) recommends antibacterial, antifungal and antiviral prophylaxis (**Table 1**). These recommendations have a low level of evidence and are not based on randomized studies conducted specifically in MM patients. Prophylaxis with cotrimoxazole, as already proven in other studies, is a practice without consensus and based on the clinical practice of each hospital (17). Cotrimixazole was associated with a reduction in *Pneumocystis jirovecii* pneumonia-related mortality (14, 15). Levofloxacin prophylaxis, based on the phase 3 TEAMM randomized trial (18), is used in clinical practice irregularly, mainly due to the emergence of quinolone resistance. A systematic review of dysglycemic effect of fluoroquinolones showed that fluoroquinolones must be used with great caution among diabetic patients who have comorbidities and are receiving antidiabetics and/or steroids and that levofloxacin was associated with a very high level of hypoglycemic diabetic patients (19). In what concerns antifungal prophylaxis, previous studies showed a drug interaction between lenalidomide and itraconazole, which is a potent inhibitor of CYP3A4 and P-glycoprotein (P-gp) activity. P-gp is involved in the lenalidomide pharmacokinetics and drug-interactions. Because lenalidomide is scarcely metabolized by cytochrome P450s, the activity of drug-transporters such as P-gp may be a key determinant of lenalidomide pharmacokinetics. As previously reported, the AUC0–24 and Cmax for lenalidomide were markedly increased by itraconazole, though its elimination t1/2 was unaffected, suggesting that the drug interaction between lenalidomide and itraconazole occurs via P-gp during absorption from the small intestine. Lenalidomide exposure could contribute to its toxicity and careful monitoring of lenalidomide as well as creatinine clearance is recommended to avoid the risk of toxicity (20). Pomalidomide is partly metabolized by CYP1A2 and CYP3A4/5 and it is also a substrate for P-gp. Concomitant administration of pomalidomide with ketoconazole, a potent inhibitor of CYP3A4/5 and P-gp, demonstrated no clinically relevant effect on pomalidomide exposure (21).

Recommendations for vaccinations in MM patients have been published by the European Myeloma Network, the CDC (Center of Disease Control), IDSA (Infectious Disease Society of America) and NCCN (National Comprehensive Cancer Network) (**Table 2**) (15, 22).

**Table 2:** Recommendations for vaccination in multiple myeloma patients .

Infections	Vaccine type	Recommendation	Doses
Influenza	Trivalent or quadrivalent	All patients, non-immune family members, close contacts and Health Care Providers	2, or 1 in case of documented seroprotection after the 1st dose, yearly
Pneumococci	PCV13 or PCV15 (followed by PPSV23) or PCV20 only	All patients with MM	1
	PPSV23	>2 months, or 6– 12 months after PCV13 or PCV15	1–3 Repeat in 3 years after 1st dose
Herpes zoster	Recombinant VZV glycoprotein E vaccine (Shingrix^®^) VZV vaccine^b^	All patients with MM	2
	(Zostavax^®^) if a recombinant vaccine is not available		4
Haemophilus influenzae	Haemophilus influenza type B conjugate	All patients with MM	1
Meningococci	Meningococcal conjugate	Patients with asplenia, recurrent episodes of bacterial infections	1
Hepatitis A	Inactivated hepatitis A vaccine	Patients traveling to areas of high endemicity	2
Hepatitis B	Recombinant hepatitis B vaccine	Patients traveling to areas of high endemicity, behavioral/occupational exposure, hemodialysis	3
SARS-CoV2 (COVID19)	Spikevax bivalent Original/Omicron BA.4- 5 Comirnaty^®^ Original/Omicron BA.4-5 Omicron attenuated vaccine variants: mRNA-1273.214 and Corminaty original/omicron BA.1 or BA.4-5	All patients with MM, non-immune family members, close contacts and Health Care Providers	2 plus a 3rd dose >4 months after the 2nd dose, plus a 4th dose 4-6 months thereafter
	Ad26.COV2, Adenovirus vector-based vaccine		2
	Novavax Protein-based		2

## Kidney injury

Kidney injury is one of the most frequent complications in MM patients, and establishing the cause is essential to the treatment approach (10). The initial evaluation should include the determination of serum urea, creatinine, electrolytes and serum free light chain (sFLC) levels, as well as urine protein electrophoresis and 24-hour urine free light chain levels. Additionally, monitoring and characterization of proteinuria is mandatory in the assessment of response to therapy and progression of MM (22, 23).

Selective proteinuria of light chains is suggestive of light chain nephropathy (LCN) and is considered not to require histological characterization, whereas non-selective proteinuria or albuminuria point to alternative causes of kidney damage (23). Renal biopsy (RB) is recommended in situations without a clear etiology, such as non-selective proteinuria, albuminuria without a prior cause or disproportionate to it, or in cases with unexplained kidney injury and sFLC < 500 mg/L. In suspected cases of AL amyloidosis, abdominal fat fine needle aspiration and bone marrow biopsy for amyloid screening should be considered, thus avoiding RB (23). While the risk associated with RB, in patients with MM overlaps that of the general population, there is an increased risk of hemorrhagic complications in patients with suspected AL amyloidosis. In these cases, minimally invasive techniques should be preferred (24).

LCN constitutes a hematological emergency, and its approach should include renal supportive measures, high-dose corticosteroid therapy, and therapy directed at the neoplastic clone (24).

Supportive measures encompass the removal of the precipitating factors of nephropathy, with vigorous hydration (2 L/m^2^/day) with particular attention to the fluid balance, especially in patients with reduced urine output and cardiac disease. Diuretics should be avoided, except in situations of fluid overload. The use of bicarbonate to achieve a urinary pH of 7 is controversial and should be avoided in hypercalcemia, due to the risk of calcium phosphate precipitation in the renal tubules (24).

Other key measures include discontinuation of angiotensin-converting enzyme inhibitors and of non-steroidal anti-inflammatory drugs, and the treatment of concomitant infections with non-nephrotoxic antibiotics (23, 24).

Hypercalcemia should be corrected urgently, with hydration and bisphosphonates, with dose-adjustment to creatinine clearance (CrCl). High-dose dexamethasone - with anti-inflammatory, catabolic and anti-neoplastic effects - should be started early, at a dose of 40 mg/day (20 mg in patients ≥75 years-old), 4 days on and 4 days off during the first month, and thereafter according to the prescribed protocol (23). In anuric patients, fluid-challenge may be attempted, with particular attention to signs of fluid overload; however, the initiation of dialysis should not be delayed in patients with severe renal failure (25).

In patients requiring dialysis, the use of high cut-off filters remains controversial, and no benefit in terms of survival or hemodialysis independence has been demonstrated in a meta-analysis of the 2 controlled studies available to date (26–28).

Therapy targeting the neoplastic clone should be initiated as soon as possible. In the absence of clear guidelines for this subgroup of patients, recommendations favor a first-line triplet regimen based on bortezomib and high-dose corticosteroid therapy (if appropriate to the patient’s frailty score), due to its efficacy, rapid onset of response, and pharmacokinetics that are independent of CrCl. According to published recommendations, bortezomib should be started on a twice-weekly basis, in association with high-dose dexamethasone (23). In patients undergoing dialysis, bortezomib should be administered preferably after dialysis. Cyclophosphamide does not require dose adjustment and the VCD regimen is recommended by the IMWG (23).

The pharmacokinetics and efficacy of carfilzomib are not influenced by renal dysfunction, making this drug a valid option at doses of 56 mg/m² (29). Ixazomib can also be used in this context, with a dose adjustment to 3 mg in cases of patients with CrCl <30 mL/min (30).

Thalidomide does not require renal dose adjustment, while lenalidomide doses should be adjusted according to CrCl. Although effective, it may be associated with increased toxicity, mainly hematological and infectious (23, 31, 32). Pomalidomide does not require renal dose adjustment and can be used in patients on hemodialysis, with comparable efficacy in patients with and without renal dysfunction and with an acceptable toxicity profile (32).

The rapid anti-neoplastic activity of anti-CD38 monoclonal antibodies allows for the potential reversibility of renal injury, and renal response rates of up to 70% have been reported with isatuximab (32).

Autologous hematopoietic stem cell transplantation (ASCT) is not universally recommended, but may be an option in selected patients. Although possible even in patients undergoing dialysis, transplant-related mortality is higher in patients with renal dysfunction (4% vs 1%) and should be considered with particular care. In these patients, melphalan dose adjustment is mandatory (100 to 140 mg/m^2^), but its efficacy seems to be preserved (23).

The selection of the therapeutic regimen in RRMM patients with renal dysfunction continues to be guided by frailty, and exposure and response to previous therapies (23, 29).

With the development of increasingly effective therapies and the increased survival of MM patients, kidney transplantation has become an option to consider in patients with dialysis-dependence and adequate response to therapy. Data from the American registry of kidney transplantation (*National Kidney Registry*) show equivalent transplanted organ lifespans in MM patients and in the general population. Survival remains lower in patients undergoing kidney transplantation due to MM, compared to other causes of renal failure, but this difference is not evident in patients over 50 years of age (33).

## Neurological manifestations

The presence of peripheral neuropathy (PN) in a MM patient should raise the suspicion of associated amyloidosis (34). Although the incidence of PN varies between authors, it is estimated that up to 20% of patients with MM may present with PN at diagnosis, and up to 75% develop this complication during therapy (35).

Treatment-induced peripheral neuropathy (TIPN) is one of the most frequent complications (35–37) with implications for the management of therapeutic protocols, patient quality of life and future therapeutic options (36).The most frequently involved drugs are thalidomide and bortezomib (38, 39). TIPN presents as a distal, symmetrical sensory polyneuropathy with associated neuropathic pain (36, 37), which occurs spontaneously or after physical stimuli, such as touch, or thermal stimuli, such as cold or heat. There may be motor involvement, usually associated with severe sensory changes, as well as autonomic symptoms such as orthostatic hypotension, bradycardia, constipation, urinary incontinence or erectile dysfunction (36, 37, 40).

Close surveillance is fundamental for the early diagnosis of TIPN, and the patient should be alerted to the main symptoms related to TIPN; the involvement of the whole patient-care team is important in monitoring its progression.

In the assessment of the patient, the use of scales based on clinical evaluation and neurophysiological studies - such as the ICT-NCN and the Total Neuropathy Score (TNS) - are important (36).

Treatment includes correction of all potentially reversible additional causes of PN and control of comorbidities [e.g. vitamin B12 deficiency, alcoholism, chronic kidney disease or diabetes mellitus (36)], as well as early dose adjustments, to limit the consequences of neurological damage and prevent irreversible changes.

Dose adjustments should be made according to the recommendations defined for each drug, and are based on the grades established by the NCI-CTC. The IMWG recommends a different schema for bortezomib, with a switch to weekly treatment in Grade 1 TIPN, and definitive discontinuation if Grade 2 or higher and with pain (35). Thalidomide should be dose-reduced by 50% if Grade 1 TIPN, and suspended if Grade 2, with resumption with a dose reduction of 50% if there is an improvement in the PN to Grade 1 (35), always taking into account the risk-benefit ratio. The duration of thalidomide therapy should not exceed 6-12 months (35, 41) Currently, the use of other active treatments against the neoplastic clone, such as other PIs (ixazomib) and monoclonal antibodies, facilitate the management of these patients.

Duloxetine has been shown to reduce pain associated with TIPN, although MM patients were not included in duloxetine clinical trials (42, 43). The use of gabapentin or pregabalin, by analogy to the benefits seen with these drugs in neuropathic pain associated with other pathologies such as diabetes mellitus, trigeminal neuralgia or post-herpetic neuralgia, is widespread (37, 44). There seems to be no advantage with pharmacological prophylaxis, and there is no solid evidence of the benefit of adjunctive practices, such as exercise, acupuncture, cryotherapy, compressive therapy or transcutaneous electrical neurostimulation (TENS). Similarly, no benefit has been demonstrated with the use of vitamins either (44).

Assessment by a neurologist and referral to a chronic pain specialist may be necessary (35).

## Cardiovascular involvement

The presence of cardiovascular (CV) risk factors or comorbidities at the time of MM diagnosis is frequent (45, 46) in elderly patients, and can be considered to fall into 3 categories:

- not directly related to MM nor to the targeted therapy, such as advanced age, diabetes mellitus, dyslipidemia, obesity, or previous cardiovascular disease
- directly related to MM, such as cardiac AL amyloidosis, hyperviscosity, high output heart failure, arteriovenous shunts, anemia, and renal dysfunction
- resulting from MM treatment-related toxicity, including through anthracyclines, corticosteroids, alkylating agents, IMIDs and PIs, often in combination regimens (47).

### Assessment of cardiovascular risk prior to the initiation of therapy

The recommendations published by the European Society for Medical Oncology (ESMO) and the European Society of Cardiology (ESC) (48, 49) regarding the management of treatment-related cardiac events indicate that an assessment of the potential risk for cardiotoxicity and of pre-existing CV risk factors - such as obesity, smoking, diabetes mellitus, hypercholesterolemia, and personal or family history of CV disease (50)- should be performed before treatment initiation, and patient education and healthy lifestyle changes should be encouraged.

Cardiac function should be assessed by objective examination, electrocardiography (ECG) and echocardiography (51) including left ventricular ejection fraction (LVEF), cardiac morphology and valvular function assessment. The basal determination of brain natriuretic peptide (BNP) and its precursor (NT-proBNP) can also be used, but there is a low specificity in MM patients (51). Some authors indicate that blood pressure (in dorsal decubitus and orthostatism) should be assessed on D1 of each treatment cycle (51). Patients treated with carfilzomib have an increased risk of cardiac toxicity after 75 years of age and if one of the following risk factors is present: heart failure with or without reduction of LVEF, coronary heart disease, valvular pathology, cardiomyopathy, uncontrolled arrhythmia or previous anthracycline therapy. Patients who are smokers, obese or sedentary are also at increased risk of cardiac adverse events (52).

Up to 30% of MM patients may have cardiac involvement by AL amyloidosis deposits, that probably will increase with improving longevity in MM. This involvement can be asymptomatic but confers dismal prognosis often constituting major morbidity. In patients with abnormal BNP/NT-proBNP values or suggestive alterations in echocardiography, a cardiac magnetic resonance should be performed (53).

### Cardiovascular risk monitoring and management during therapy

During treatment, patients and physicians should be aware of possible signs and symptoms of CV origin. Symptoms of dyspnea, chest pain, edema or fatigue should be investigated to ascertain whether they are being caused by cardiac dysfunction. Monitoring of cardiac function during treatment using echocardiography and ECG is also recommended. If a significant decline in LVEF is observed (>10% below the lower limit of normal), initiation of targeted treatment for left ventricular dysfunction with angiotensin-converting enzyme inhibitors and beta-blockers should be considered (48). The decision to continue or discontinue treatment must be made individually, taking into account the context of each patient, their relative risk, overall CV status and also the probability of cardiac effects of the prescribed drugs (51). In the case of treatment with carfilzomib, the recommendations of the European Myeloma Network (EMN) for grade 3 (or higher) cardiac adverse events are to suspend the drug and administer fluids. If deemed appropriate, carfilzomib therapy can be restarted at a lower dose level, based on an individualized risk-benefit assessment. A sub-analysis of the ENDEAVOR study suggests that the reduction in LVEF related to bortezomib or carfilzomib is in most cases reversible (54). When therapy is restarted, follow-up should be considered with regular echocardiography (52) and clinical evaluation, whenever possible in a cardio-oncology consultation.

Prevention remains crucial to improve patient care in this setting. Cardioprotective drugs can be given in addition to specific thromboprophylaxis whenever necessary. There are a small number of studies suggesting that angiotensin-converting enzyme inhibitors such as enalapril, angiotensin receptor blockers such as candesartan, and selected beta-blockers such as carvedilol and nebivolol, may be the preferred agents to reduce the risk of cardiotoxicity, in addition to mineralocorticoid receptor antagonists such as spironolactone (55).

## Hemostatic abnormalities

### Thrombotic Complications

The incidence of venous thromboembolism (VTE) in patients with MM is approximately 9 times higher than in the general population, with about 8-12 cases per 1,000 patient-years; the risk of arterial thrombosis is also increased. VTE is a leading cause of death in cancer patients and, in the case of MM patients, the presence of VTE in the first year after diagnosis is associated with a three-fold increase in the risk of death, compared to patients without VTE (56). In addition to the increased risk induced by the neoplasm, some drugs (e.g. IMIDs) specifically increase the risk of thromboembolism. Several scores have been published to stratify the risk of VTE (and the consequent indication for prophylaxis) in patients with MM (IMWG, SAVED, IMPEDE VTE); however, none has demonstrated a satisfactory discriminatory power, and this remains an unmet need (56–58).

Increased D-dimer and PAI-1 levels have been associated with an increased risk of VTE in MM patients. However, elevation of D-dimer above the normal upper limit was nearly universal with no consensus on a clear cut-off point at which it becomes predictive. Data about fibrinogen, FVIII, alpha-2-antiplasmin and Protein C and S found no significant relation with VTE risk and markedly conflicting results were observed to date. Also, the toxic effect of anti-myeloma therapies on the endothelium and contribution to thrombosis has been widely described (59, 60).

VTE therapy in patients with MM should be carried out according to international guidelines (61, 62). Before initiation of therapy with IMIDs, risk factors for VTE should be assessed (**Table 2**). The guidelines published by the IMWG (2008), still widely used today, advocate for the use of acetylsalicylic acid (ASA) 75-325 mg od in patients with 0 to 1 risk factors, and low molecular weight heparin (enoxaparin 40 mg od, or equivalent) or warfarin for a target INR of 2.0-3.0, in patients with 2 or more risk factors (63)(**Table 3**) and all those receiving high dose dexamethasone (≥480 mg per month), concomitant doxorubicin or multidrug regimens (excluding bortezomib).

**Table 3:** Risk factors for venous thrombosis in patients with multiple myeloma.

• Obesity (Body Mass Index ≥30 kg/m2) • Previous venous thromboembolism • Central venous catheter • Pacemaker • Heart disease • Chronic renal disease • Diabetes mellitus • Acute infection • Immobilization • Recent surgery (less than 6 weeks) • Trauma • Thrombophilia • Therapy with erythropoiesis-stimulating agents

The guidelines recently published by the American Society of Hematology (ASH), recommend the use of low-dose ASA, low and fixed-dose vitamin K antagonists (1.25 mg daily) or low molecular weight heparin (LMWH) in patients receiving regimens including IMIDs (61). Despite its superior efficacy, especially in high-risk patients, the use of subcutaneous (SC) therapy with LMWH for prolonged periods becomes a less comfortable option in the long term due to the route of administration (61).

The evidence for the direct oral anticoagulants (DOACs) in VTE prophylaxis in MM and cancer, in general, is still limited (with some studies demonstrating efficacy at the cost of an increased risk of bleeding, and with most trials including <5% of MM patients). Several trials with DOAC are ongoing, and many authors already advocate their use, based on their safety profile and convenient administration (64–67).

According to ASH recommendations, therapy with LMWH or with DOACs (apixaban and rivaroxaban) is indicated in the first 5-10 days after a thrombotic event. In patients with severe renal impairment (CrCl <30 mL/min), non-fractionated heparin should be considered, although from a practical point of view, this option is less feasible. Over the subsequent 3 to 6 months, therapy with a DOAC (apixaban, edoxaban, or rivaroxaban), LMWH or, as a last resort, warfarin, is recommended. In patients with CrCl <30 mL/min, warfarin is the preferred option for prolonged therapy. For patients with recurrent VTE despite LMWH at a therapeutic dose, recommendations suggest increasing the dose to supra-therapeutic levels or maintains a therapeutic dose (61). In patients with active cancer and VTE, long term maintenance of anticoagulation at a therapeutic dose is suggested. The choice of NOACs over vitamin K antagonists is also recommended in the guidelines of other scientific societies (61, 62).

Thrombocytopenia is a frequent constraint in VTE prophylaxis. Prophylaxis should be maintained, with close monitoring, in patients with platelet counts of 50 to 100 x 10^9^/L,and discontinued in the majority of patients with platelet counts <50 x 10^9^/L, particularly after 6 months of therapy with IMIDs (where the risk of thrombosis is lower); maintained could be indicated in high-risk patients, always weighing thrombotic and bleeding risks (68).

### Hemorrhagic complications

Hemorrhage is rarely associated with MM at diagnosis, being mostly prevalent in progressive disease, particularly in association with hyperviscosity syndrome, thrombocytopenia, renal failure or infection (68, 69).

Abnormal screening coagulation test results, including prothrombin time (PT), activated partial thromboplastin time (APTT), and thrombin time (TT), are commonly encountered in patients with plasma cell neoplasms and are not typically associated with clinically significant bleeding (69).

In patients on antithrombotic prophylaxis and IMIDs, the incidence of major bleeding is higher in patients with a previous history of bleeding, renal disease or advanced age (70).

The use of ASA in combination with corticosteroids (particularly dexamethasone) is associated with an increased risk of bleeding, especially gastrointestinal hemorrhage, so caution is advised in patients with previous episodes of ulcerative peptic disease, or other causes of digestive bleeding (61).

## Bone disease

Bone disease, in particular lytic lesions, result from the neoplastic proliferation of plasma cells and alterations in the bone remodeling mechanisms, leading to bone pain. The management of bone pain in MM is mostly palliative but essential for the patient’s quality of life. It includes antiresorptive therapy (e.g. bisphosphonates), to prevent new lesions, radiotherapy, surgery, corticosteroids and analgesia (71).

Intravenous antiresorptive therapy with bisphosphonates should be administered in MM patients under chemotherapy, regardless of the existence of bone disease, although its benefit in patients without bone disease staged by MRI or PET has not been unequivocally demonstrated (71).

Zoledronate or pamidronate, at 28-day intervals, should be given for at least 12 to 24 months in patients with a complete response or a very good partial response, and until disease progression in the case of partial response or stable disease (71). Patients should be monitored for renal function and electrolyte imbalance – particularly of calcium and phosphorus -, and 600 mg of calcium and 400 IU of vitamin D supplements should be given daily.

Osteonecrosis of the jaw (the exposure of jaw bone without healing after 2 months of therapy) is a complication described in 5-10% of patients on bisphosphonate therapy, with an incidence of 1% per year (68). Risk factors include dental procedures, local infections or concomitant corticosteroid therapy. Dental evaluation is recommended before bisphosphonate therapy is started, with dental extractions or other more invasive procedures performed, if necessary. If there is an episode of osteonecrosis, there is no contraindication to restarting bisphosphonates after healing (68).

Radiotherapy is an excellent tool to consider for the relief of bone pain, particularly in acute pain occurring in the context of vertebral collapse. A single dose of 8 Gy is recommended (68). Higher doses, up to 30 Gy, fractionated over 2 weeks, are associated with better pain control and should be considered when there is an associated soft tissue component or pathological fracture with spinal cord compression (72).

Vertebroplasty and kyphoplasty are alternatives to control pain associated with vertebral collapse. These techniques consist of the percutaneous injection of bone cement to stabilize the vertebral body, and should ideally be performed as soon as possible after vertebral collapse. Both techniques carry a low risk of extravasation of bone cement, with the possibility of neurological compromise or thromboembolism (71, 73). These techniques are equally effective as surgery in treating pain, with very low infectious risk (74). If vertebral collapse with spinal cord compression occurs, this must be approached as a medical emergency, due to the risk of irreversible neurological lesions.

## Chronic and acute pain

Chronic pain is one of the most frequent symptoms in MM patients, in relation either to bone disease or to peripheral neuropathy. The WHO establishes a 3-step algorithm in pain management, taking into account the pain score reported by the patient:

- Scores 1-4: do not require opioid use. Paracetamol (up to 3 g/day, oral or IV) should be preferred. Non-steroidal anti-inflammatory drugs should be used with caution due to their renal, gastrointestinal and hematological side effects.

- Scores 5-6: weak opioids associated with non-opioids, such as tramadol (up to 400 mg/day, per os, SC, IM or IV) and paracetamol, may be used.

- Scores 7-10: strong opioids are mandatory. Morphine (oral, SC or IV, including continuous infusion) is a good option given its efficacy, safety profile, versatility, and low cost. Fentanyl is about 100 times more potent than morphine and, although it can be given IV, it is primarily given transdermal in chronic pain, and via the transmucosal or sublingual route in acute pain (75).

Patients with pain scores greater than 5 or who, after analgesic therapy, do not show a score reduction of at least 2 points, should be referred to pain medicine teams (68).

The most common adverse effects of opioids include constipation, nausea and vomiting, pruritus, urinary retention, respiratory failure, and tolerance and hyperalgesia (with these latter two situations requiring referral to pain medicine specialists). Concomitant prescription of antiemetics and laxatives is advised (68, 75).

Given the mixed etiology of pain in MM patients, a multimodal approach is the most appropriate. In this regard, the concomitant use of calcium channel blockers such as gabapentin or pregabalin, as well as antidepressants such as duloxetine or amitriptyline should be considered (76).

## Metabolic disorders

### Hyperglycemia

Diabetes is a frequent comorbidity of MM, with an estimated 11-22% of MM patients presenting with diabetes or other disorders of glucose metabolism (8). It is recommended that MM patients be screened for diabetes (by fasting blood glucose and, ideally, HbA1c levels) before initiation of corticosteroid therapy, particularly if they have additional risk factors such as age, obesity, a family history of diabetes or a personal history of gestational diabetes (8, 77).

Patients previously on oral antidiabetics who maintain adequate glycemic control should maintain their usual therapy. However, most patients will need to initiate insulin therapy, and those previously on insulin will likely need to increase basal and pre-prandial insulin doses 2- to 3-fold (8, 77). On the other hand, it should be noted that patients with MM often present with anorexia and nausea/vomiting, which can lead to hypoglycemic events, particularly when medicated with high-risk oral antidiabetics, such as sulfonylureas, or high doses of basal insulin; basal therapy adjustments are recommended, particularly by changing sulfonylureas to metformin and/or glinides, as well as avoiding mixed insulins and favoring rescue with rapid insulin according to pre-prandial glycemic levels (8, 77). Reductions in the dose of corticosteroids must be accompanied by a reduction in insulin doses, to minimize the risk of hypoglycemia (77).

In addition to hyperglycemia, diabetes mellitus is associated with multiple organ dysfunctions - neuropathy, nephropathy, retinopathy and cardiovascular changes - which may also be induced by MM itself, worsening their severity and potentially confounding an adequate diagnosis and a timely correction (8).

### Electrolyte imbalances: hyper- and hypocalcemia

The mainstay of therapy for hypercalcemia is intravenous hydration (0.9% sodium chloride, 35 mL/kg/day in young patients, and 15-20 mL/kg/day in elderly patients with renal, hepatic or cardiac dysfunction), bone disease-modifying agents, calcitonin, corticosteroids and, in particularly severe and refractory cases, hemodialysis. Diuretics should be delayed until adequate hydration has been ensured, and calcium and vitamin D supplements should be discontinued (64, 72, 78).

Prevention of bone resorption with targeted therapies (namely bisphosphonates) is essential to restore the homeostasis of calcium metabolism, with an onset of action about 2-4 days after administration (79). Among the bisphosphonates, zoledronic acid has been shown to be superior in the rate of normalization, time to resolution and duration of hypercalcemia control, compared with pamidronate (80). Zoledronic acid is administered in a single dose of 4 mg (in 100 mL of 0.9% sodium chloride or 5% glucose, infused over 15-30 minutes) in patients with a corrected serum calcium ≥ 12 mg/dL, whereas the maximum daily dose of pamidronate is 90 mg (diluted in 500 mL of 0.9% sodium chloride or 5% glucose, given in a 2-4 hour infusion, at a maximum rate 1 mg/min), once every 4 weeks. A supplementary dose may be given in the absence of reversal of hypercalcemia after 3-7 days (81).

Dose adjustment of bisphosphonates is necessary in renal dysfunction (**Table 3**) (72, 79), and bisphosphonates are formally contraindicated in severe renal dysfunction (CrCl <30 mL/min); however, in the case of severe hypercalcemia in which the benefits outweigh the risks, the lowest dose should be given, with a prolonged infusion time. In the case of moderate renal impairment, the dose should be adjusted according to **Table 4** (82).

**Table 4:** Dose and infusion time of bisphosphonates according to renal function.

Bisphosphonate	Dose	Infusion time
Zoledronic acid	CrCl > 60 mL/min – 4.0 mg CrCl 50-60 mL/min – 3.5 mg CrCl 40-49 mL/min – 3.3 mg CrCl 30-39 mL/min – 3.0 mg CrCl <30 mL/min – not recommended*	15-30 min
Pamidronate	CrCl >60 mL/min – full dose (90 mg) CrCl <30 mL/min – not recommended*	2-4 hours (CrCl >60 mL/min; maximal infusion rate 1mg/min). 4 hours (CrCl 30-60 mL/min) 4-6 hours (CrCl <30 mL/min)

CrCl, creatinine clearance.

*May be considered if severe and symptomatic hypercalcemia.

Denosumab, a humanized monoclonal antibody directed to the RANK ligand (receptor activator of nuclear factor Kappa B) may be considered in the management of hypercalcemia that is refractory to bisphosphonates, as well as in patients with severe renal dysfunction, as it is not excreted by the kidneys (79). Denosumab has been shown to be associated with a greater reduction in serum calcium levels when compared with zoledronic acid (83). The recommended dose in hypercalcemia is a single administration of 120 mg, sc, in patients with serum calcium levels >12.5 mg/dL (79).

Calcitonin can be administered at a dose of 4-8 IU/kg, sc, every 6-12 hours, with a faster onset of action than bisphosphonates (within the first 4-6 hours after administration). However, its effect is limited in time (24-48 hours) due to the risk of tachyphylaxis (64, 84).

Although MM is classically associated with hypercalcemia, hypocalcemia may be present, mostly as a consequence of hypercalcemia therapy or bone disease. It is almost always asymptomatic; symptoms (such as paresthesia, cramps and rarely cardiac dysfunction) usually are only present with severe hypocalcemia (serum calcium levels <7 mg/dL) (64, 82).

Treatment-induced hypocalcemia is more frequently seen in patients on denosumab therapy (17%) than in those on zoledronic acid (12%) (83). In patients with renal dysfunction, denosumab dose adjustments should be considered due to the increased risk of hypocalcemia (85).

### Tumor lysis syndrome

MM is associated with a low incidence of tumor lysis syndrome (TLS); patients at higher risk are those with baseline hyperuricemia, renal dysfunction, volume depletion or high tumor burden. In the current therapeutic scenario, bortezomib and carfilzomib are the drugs most frequently associated with this complication. Prophylaxis of TLS is recommended with reinforcement of oral hydration and allopurinol (100-300 mg/day) (64, 86, 87). Anti-myeloma monoclonal antibodies (daratumumab, isatuximab, elotuzumab) and the antibody-drug conjugate belantamab mafodotin have been shown to induce TLS (88), as has CAR-T cell therapy (89).

In patients with ongoing TLS or severe hyperuricemia, administration of rasburicase (0.2mg/kg/day, in 50 mL of 0.9% sodium chloride, infused over 30 minutes) is recommended (64, 86).

## Neuropsychiatric symptoms

Corticosteroids are associated with a wide spectrum of clinical manifestations - ranging from sleep and mood disturbances, including insomnia, irritability, depression and euphoria, to cognitive impairment, psychosis and delirium -, in about 60% of patients. Although existing research suggests that there is a dose-dependent relationship between steroids and neuropsychiatric symptoms, with increased cases reported at higher doses, even very low doses can lead to symptoms (90, 91).

Corticosteroid-induced neuropsychiatric symptoms have a variable chronological relationship to the initiation of therapy, ranging from hours from the first administration, to late manifestations and even after the terminus of therapy. However, most manifestations occur early after the start of therapy (days) (90).

Among the corticosteroids, those with a long duration of action (such as dexamethasone) are associated with a higher incidence of symptoms, and evidence suggests that non-continuous regimens may have less severe symptoms (90).

The hyperviscosity syndrome (present in 2-6% of patients with MM) also manifests itself with non-specific neurological symptoms that range from the - headache, dizziness, tinnitus, difficulty concentrating, sleepiness or hypoacusis to ataxia, convulsions, and even coma, in addition, to an increased risk of stroke (92).

In the presence of persistent unexplained psychiatric and neuropsychiatric symptoms, particularly in patients who have previously undergone multiple lines of therapy, it is important not only to perform central nervous system (CNS) imaging (with CT and/or MRI) but also a cerebral spinal fluid analyses to exclude CNS involvement by MM (present in <1% of patients and usually in progressive disease) or infection (bacterial, viral or fungal meningitis, or more rarely infection by the JC virus) (64, 93).

## Ocular disorders

Ocular manifestations due to the direct invasion of ocular structures by MM are rare, and may be present in heavily pretreated patients and with uncontrolled systemic disease. Therapy focuses on systemic treatment, targeted radiotherapy and, in specific cases, decompressive surgery (93, 94).

Hyperviscosity syndrome can present with visual manifestations, such as blurred vision or diplopia. Ophthalmoscopic examination of the fundus can reveal characteristic findings of hyperviscosity, such as the presence of dilated and tortuous retinal veins, flame-shaped hemorrhages, edema of the papilla, and retinal exudates. Although uncommon, there may be central retinal vein occlusion and retinal detachment (92).Once the diagnosis is established (or if suspicion is high), emergent therapy with plasma exchange is imperative. Initial exchange of 1.5 plasma volumes in the first session and 1 volume in subsequent sessions is recommended. Replacement fluids should be 5% albumin and fresh frozen plasma, to reduce the risk of coagulopathy. Although there is no evidence regarding the ideal number of sessions, daily sessions for three days with frequent reassessment and simultaneous initiation of MM-targeted therapy are recommended.

It Is estimated that about 12% of MM patients experience ocular adverse effects of treatment, with the most common being keratopathy/keratitis, xerophthalmia and blurred vision. In most cases, there is a resolution with topical therapy (95).

In addition, some of the drugs used in MM treatment have specific ocular toxicity. Glaucoma and cataracts are the main adverse effects of corticosteroids. In the case of glaucoma, therapeutic protocols with lower doses of corticosteroids should be considered (96). Patients at high risk for cataracts (namely those with a previous history of glaucoma) and/or those with reduced visual acuity should be observed by the ophthalmologist (97).

Corneal epithelium changes (keratopathy) were found in patients treated with belantamab mafodotin, an antibody-drug conjugate targeting the B-cell maturation antigen (BCMA), in the DREAMM-2 study, where it led to the majority of dose adjustments, treatment delays and discontinuations. Patient education is key to minimizing and preventing complications associated with belantamab mafodotin. To minimize the risk of ocular toxicity, it is imperative that patients see an eye specialist for baseline assessment prior to starting treatment and prior to each subsequent dose, to monitor for worsening eye symptoms (dry eyes, blurred vision, deteriorating visual acuity, and corneal ulceration). To help reduce the incidence of ocular events, patients should be educated on the use of preservative-free ophthalmic lubricants at least four times daily starting prior to the first treatment and continuing to the end of treatment. Since patients with preexisting corneal disease are at higher risk of developing keratopathy, contact lens use discontinuation is recommended while on therapy (98).

## Cutaneous lesions

Exanthema is one of the most common non-hematological adverse effects of IMIDs and PIs (although less frequent and less severe with the latter), and may present different features including morbilliform, dermatitis-like, acneiform or urticarial exanthema. Mild to moderate cases of exanthema associated with PI therapy or IMIDs can be treated with topical corticosteroids and oral antihistamines, while in severe cases discontinuation of the drug and the use of systemic corticosteroids are recommended. There is usually good tolerance to the reintroduction of the drug (particularly lenalidomide) within 1-2 weeks of resolution, and desensitization protocols can be performed in patients with recurrence of symptoms (99–102).

## Gastrointestinal manifestations

Gastrointestinal tract symptoms are very frequent in patients with MM (103, 104). Constipation is a complaint often associated with opioid analgesics but also with thalidomide and 5-HT3 receptor antagonists (used in the prevention or treatment of emesis) (68). Dietary measures and laxatives are useful in these situations (68, 105). Diarrhea is also a symptom frequently associated with bortezomib and ixazomib (in up to 45% of patients) (106) and occurs on the day of administration or the following day (107). Therapy is symptomatic; in severe cases, dose reduction or even suspension of these drugs may be necessary (106).Lenalidomide-associated diarrhea is also of note, as it is frequent with prolonged use (occurring in about 30% of patients) (105, 108). It appears several months after the start of treatment (108) and seems to be related to bile acid malabsorption (109). Reduced dietary fat intake and the use of bile acid chelators such as cholestyramine are recommended (109). General measures in any patient with diarrhea include proper hydration, a low-fiber diet, and the consumption of frequent, lighter meals. Loperamide and electrolyte replacement therapy may also be used (105).

Several treatment protocols include a prolonged use of corticosteroids, which can lead to an increased risk of gastrointestinal bleeding and colonic perforation (110, 111). Therefore, prophylactic proton pump inhibitors should be prescribed concomitantly with these drugs.

Taking into account that gastrointestinal symptoms are very frequent and non-specific and that, although they may be related to the disease, to treatment toxicity, or to other causes such as infections, neoplasms and AL amyloidosis (112), careful investigation is recommended, with evaluation by a gastroenterologist whenever necessary.

## Second primary malignancies

The risk of second primary malignancies (SPM) in MM is low and is partially related to the increased survival of these patients (113), and no particular oncological screening program has been defined within this context. The development of SPM is multipronged, including treatment-related factors (such as alkylating agents or lenalidomide), MM-related factors (with an increased risk of acute myeloid leukemia in IgG and IgA gammopathies), patient-related factors (genetic polymorphisms, advanced age, male gender, or previous neoplasms), as well as environmental and behavioral factors (114).

Combined exposure to drugs such as lenalidomide and oral melphalan appears to be the main factor associated with a substantially increased risk of hematological SPM (acute myeloid leukemia and myelodysplastic syndrome) (113, 115). Some studies also evidence an increased risk (although lower) of SPM in patients treated with lenalidomide on a maintenance regimen (113). However, this risk is largely outweighed by the favorable impact of maintenance therapy on patient survival.

The most frequent SPM include cutaneous neoplasms (basal cell or spinal cell carcinoma) (114), solid neoplasms, mainly urinary tract tumors (partly explained by the renal excretion of lenalidomide) (113) and hematological neoplasms. An increased incidence of SPM has not been observed with the association of lenalidomide and dexamethasone, or with the various associations with bortezomib (melphalan, dexamethasone, or thalidomide) (113).

Thus, the therapeutic decision process in MM should not influenced by the risk of SPM, since the risk of death from MM is substantially higher than the risk of death from a SPM. However, and because of the above, treatment regimens including associations of lenalidomide with melphalan should be avoided. Furthermore, close and timely monitoring of more susceptible organs or systems is fundamental to the early diagnosis of a SPM.

## Teratogenic risk

Due to the low incidence of MM in young adults, the available data regarding the safety of anti-myeloma drugs during pregnancy are very limited. First-generation alkylating drugs and PIs, such as bortezomib, are among the drugs most often used in the treatment of MM, and have been associated with a risk of intrauterine death and fetal malformations in animal studies. For this reason, the FDA classifies these drugs in category D of pharmacological risk in pregnancy (116, 117). Corticosteroids are the group of drugs with the best safety profile when used during pregnancy, especially after the first trimester (category C pharmacological risk in pregnancy, according to the FDA (118, 119). Data on the use of monoclonal antibodies and subsequent-generation PIs in pregnant women are insufficient, so their use is not recommended during pregnancy or in women of childbearing potential who do not use contraception (116, 119) during treatment. IMIDs are contraindicated during pregnancy because of their well-established teratogenic risk (113, 119).

## Toxicities associated with autologous stem cell transplant

In the era of innovative drugs, significant progress has been achieved in the treatment of multiple myeloma. However, for young and eligible patients, high-dose chemotherapy with melphalan followed by autologous stem cell transplant (ASCT) continues to stand as the gold standard. This approach boasts a remarkably low transplant-related mortality rate, ranging from 1% to 2%, despite the rapid evolution of treatment modalities (120, 121).

Over the past few decades, advancements in hematopoietic stem cell mobilization have given rise to the creation of regimens that are both less toxic and more effective. Notably, there are now chemo-free protocols, such as the combination of G-CSF plus plerixafor, which eliminate the concerns associated with neutropenia and the potential risk of febrile neutropenia (122).

Hematological toxicity is an anticipated side effect that impacts every multiple myeloma (MM) patient undergoing autologous stem cell transplant (ASCT), albeit with varying degrees of severity. Following the myeloablative effect induced by melphalan and occurring prior to engraftment, typically around Day 12 post-transplant, patients experience profound neutropenia and lymphopenia, along with varying levels of anemia and thrombocytopenia (121). During this period, febrile neutropenia is highly prevalent, affecting approximately half of patients, particularly those with severe oral and gastrointestinal mucositis, reaching grades 3-4 in 30 per cent of cases (123).

Non-pharmacological interventions for oral and gastrointestinal mucositis, as well as nausea/vomiting, such as cryotherapy and modified diets, respectively, can prove beneficial in alleviating patients’ distress and reducing the severity of symptoms (121, 124, 125).

Prophylaxis for HSV/VZV using acyclovir, for Pneumocystis jirovecii with trimethoprim-sulfamethoxazole, and antifungal with fluconazole should ideally be administered until Day 100 post-transplant (123, 126).

A delay in the recovery of lymphocyte count and function, which can extend up to 2 years for CD4+T cells, contributes to an elevated risk of infections beyond Day 100 post-transplant. This heightened susceptibility is primarily attributed to encapsulated bacteria, viruses, Aspergillus spp., and Pneumocystis jiroveci. The myeloablative conditioning regimen results in the loss of immunological memory, necessitating patients to essentially repeat the entire vaccination plan (123, 126).

High-dose melphalan conditioning does not appear to elevate the risk of secondary neoplasms. The 10-year incidence rates for secondary myelodysplasia and acute myeloid leukemia stand at 4% and 2%, respectively (121, 127).

## Toxicities associated with T-cell engaging therapies

In recent years, immunotherapy has made significant strides in the field of multiple myeloma, particularly with the introduction of T-cell engaging therapies, including bispecific T-cell engagers (BiTEs) and chimeric antigen receptor (CAR)-T cells. Currently, both the FDA and EMA have granted approval for BiTEs such as teclistamab (anti-BCMA/CD3), talquetamab (anti-GPRC5D), and elranatamab (anti-BCMA/CD3), as well as anti-BCMA CAR-T cells, namely ciltacabtagene autoleucel and idecabtagene vicleucel (128, 129).

However, these therapies brought along additional toxicities, requiring the adoption of an entirely new approach (130, 131).

### Cytokine release syndrome (CRS)

The cytokine release syndrome (CRS) is a multisystemic syndrome observed in both BiTEs (72-56%; <1% grade≥3) and CAR-T therapies (84-95%; 4-5% grade≥3). It arises from T-cell activation and proliferation, resulting in systemic inflammation (131).

In patients receiving BiTEs, CRS typically manifests during step-up doses and the initial full dose. Conversely, in CAR-T cell therapy, it generally occurs 1-14 days post-infusion with varying durations (131, 132).

CRS is characterized by fever accompanied by hemodynamic instability and/or hypoxemia, with severity graded according to the American Society for Transplantation and Cellular Therapy consensus criteria (Hayden et al., 2022). A high level of suspicion is crucial for an appropriate approach, emphasizing the importance of early diagnosis and therapy. Management of CRS is contingent on its severity, incorporating supportive therapy (IV fluid hydration, vasopressor support, supplemental oxygen/mechanical ventilation, and antibiotics). Additionally, tocilizumab (IL-6 receptor antagonist), corticosteroids, and, in refractory cases, siltuximab (anti-IL-6) or anakinra (anti-IL1-R) are used (131–133).

### Immune effector cell-associated neurotoxicity syndrome (ICANS)

The immune effector cell-associated neurotoxicity syndrome (ICANS) encompasses a range of neurological symptoms, often emerging within the initial days following CAR-T infusion (18-17%; 2-3% grade≥3) or during the administration of the first BiTEs doses (3-6%; 0-1% grade≥3). Despite ongoing research, the precise cause of ICANS remains incompletely understood, with various studies proposing that its pathophysiology revolves around the release of cytokines and subsequent inflammation in the central nervous system (131, 132).

Manifestations of ICANS span from mild symptoms like tremors, somnolence, or dysgraphia to more severe conditions such as aphasia, apraxia, seizures, or even coma. Monitoring and grading involve the systematic use of the 10-point Immune Effector Cell Encephalopathy (ICE) scale (132). A comprehensive diagnostic approach, including consultation with a neurologist, electroencephalogram, and MRI evaluation, is crucial. Treatment is centered on a symptomatic approach utilizing antiseizure drugs, corticosteroids, and, in severe cases, siltuximab or anakinra (131–133).

Delayed neurotoxicity, primarily observed in CAR-T cell therapy (affecting 12% of patients), may present as cranial nerve palsies, neuropathy, or parkinsonism. Treatment is based on corticosteroids and intravenous immunoglobulin (131, 132).

### Infectious complications

One of the most prevalent complications involves opportunistic infections, primarily stemming from neutropenia, lymphopenia, and B-cell aplasia, leading to hypogammaglobulinemia. As a result, prophylaxis is necessary until immune reconstitution (132). Consensus guidelines advocate for acyclovir/valacyclovir to prevent HSC/VZV reactivation, trimethoprim-sulfamethoxazole for Pneumocystis jirovecii (in CAR-T cells: recommended for 12-18 months post-infusion, at least until CD4 count exceeds >200/μL; in BiTEs: during treatment and up to 1 month post-treatment discontinuation), and antifungal prophylaxis with fluconazole during periods of prolonged severe neutropenia or extended steroid therapy (131, 134).

Following CAR-T cell therapy (preceded by lymphodepletion), neutropenia and lymphopenia are common, impacting approximately 10-20% and 40-50% of patients, respectively, within 3 months post-infusion. Bacterial and respiratory viral infections dominate in the first month, transitioning to a prevalence of viral infections thereafter (132, 134). Particularly in T-cell engaging therapies targeting BCMA, hypogammaglobulinemia stands out as a key risk factor for infections. Therefore, the replacement of immunoglobulins is recommended for cases with gammaglobulin levels <4 g/L, particularly when associated with serious, recurrent, or chronic infections (131, 132, 134).

### Other toxicities

Hematological toxicity represents a frequent adverse event associated with T-cell engaging therapies, particularly CAR-T cells. The incidence and severity of this toxicity tend to escalate in patients experiencing complications such as CRS, ICANS, or immune effector cell-associated hemophagocytic lymphohistiocytosis-like syndrome (IEC-HS)/macrophage activation syndrome (MAS). In such cases, cytopenias can be profound and prolonged, heightening the vulnerability to infections. The management of this toxicity aligns with established approaches for other drugs, involving interventions like transfusions and growth factor support, primarily with G-CSF. However, a small subset of refractory patients may necessitate more advanced measures, such as autologous hematopoietic stem cell boost or allogeneic hematopoietic cell transplantation (130–132, 135).

Certain agents exhibit distinct and common adverse events, as observed with talquetamab, which is known to frequently induce skin and nail-related events, along with dysgeusia (131).

## Conclusions

Despite the increased efficacy and survival with new treatment options, patients with MM continue to present with a high burden of symptoms, resulting not only from the disease but also from treatment toxicities, even in periods of disease remission and off treatment. These symptoms and adverse events have a profound impact on their quality of life, namely due to sequelae of bone destruction, reduced physical capacity and loss of muscle mass, frequently associated with chronic pain, therapeutic toxicities and worsening of comorbidities. Thus, personalization of treatment in MM patients is critical to ensure effective long-term disease control and should be based on individual patient factors such as age, general condition, comorbidities, and side effects of previous treatments. Rapid and appropriate intervention on treatment-related adverse events and on worsening of comorbidities should be based on scientific evidence, consensus recommendations, clinical experience, and is of extreme importance for patients and their families, impacting the prognosis and quality of life of those who suffer daily with MM.

## Author contributions

CG: Conceptualization, Methodology, Supervision, Writing – original draft, Writing – review & editing. AR: Methodology, Writing – original draft, Writing – review & editing. ABSR: Methodology, Writing – review & editing. MN: Methodology, Writing – original draft, Writing – review & editing. AI: Methodology, Writing – original draft, Writing – review & editing. RG: Methodology, Writing – original draft, Writing – review & editing. AT: Methodology, Writing – original draft, Writing – review & editing. SA: Methodology, Writing – original draft, Writing – review & editing. MS: Methodology, Writing – original draft, Writing – review & editing. PS: Writing – review & editing. RB: Conceptualization, Methodology, Supervision, Writing – original draft, Writing – review & editing. CJ: Conceptualization, Methodology, Supervision, Writing – original draft, Writing – review & editing.
